# Cyclical iron supplementation to reduce anemia among Brazilian preschoolers: a randomized controlled trial

**DOI:** 10.1186/1471-2458-13-21

**Published:** 2013-01-10

**Authors:** Geraldo GPL Coutinho, Patrícia M Cury, José A Cordeiro

**Affiliations:** 1 Department of Post graduation, School of Medicine, São José do Rio Preto, São Paulo 15090-000, Brazil; 2 Department of Pathology and Forensic Medicine, School of Medicine, São José do Rio Preto, São Paulo, 15090-000, Brazil; 3 Department of Epidemiology and Statistical Analysis, School of Medicine, São José do Rio Preto, São Paulo, 15090-000, Brazil; 4 Departamento de pós-graduação, Faculdade de Medicina de São José do Rio Preto, Av. Brigadeiro Faria Lima, São José do Rio Preto, SP, 5416 ZIP 15090-000, Brazil

**Keywords:** Iron, Anemia, Supplementation, Brazil, Preschoolers

## Abstract

**Background:**

Iron-deficiency anemia is the most common type of nutritional disorder. New strategies for the treatment of anemia are very important for its reduction. The aim of this study was to assess the efficacy and feasibility of cyclical iron supplementation as a strategy to reduce the prevalence of anemia among preschoolers.

**Methods:**

A randomized controlled trial was performed in the entire population of under five-year-old children who attended government daycare centers in a small town in the State of Sao Paulo, Brazil. The children were randomly allocated into two intervention groups: the Weekly and Cyclical Groups. During a ten-month period, the Weekly Group (n = 51) received weekly doses of 30 mg elemental iron (40 doses) and the Cyclical Group (n = 48) received two cycles of 20 daily doses of 30 mg elemental iron separated by a four-month period (40 doses).

**Results:**

Overall, at the end of ten months, the prevalence of anemia of the children on both supplementation regimens showed a significant decrease from 20.20% to 5.05% (p-value < 0.0005). There was no significant difference in the anemia between the two groups (p-value = 0.35). The mean hemoglobin concentration increased by 0.27 g/dL (p-value < 0.016) and 0.47 g/dL (p-value < 0.0005) in the Weekly and Cyclical Groups, respectively; again there was no significant difference between groups (p-value = 0.17). However, the cyclical regimen was easier to manage.

**Conclusions:**

Both supplementation regimens significantly reduced the prevalence of anemia however administration of the Cyclical Group was easier to carry out and control.

**Clinical trial registration number:**

NCT00992823

## Background

Iron-deficiency anemia is the most common type of nutritional disorder. It affects populations around the entire World but in particular those of poorer communities in developing countries [1]. The two most vulnerable groups are preschool children and pregnant women [1]. While anemia is recognized as a serious public health problem worldwide, its prevalence remains at unacceptably high levels [2]. Although there are few recent data on the prevalence of anemia in Brazil, studies in the State of Sao Paulo report prevalences of between 30% and 69% depending on the type of community studied [3-5].

Guidelines for treating preschoolers, as presented by the United Nations Administrative Committee on Coordination/Sub-Committee on Nutrition (ACC/ASN) [6], recommend supplementation in daily doses of elemental iron for two to three weeks several times each year. Thus, this guideline is vague as although the regimen should be repeated ‘several times each year’, the ideal interval between supplementations is not defined. The result is that some health departments repeat the regimen too few times and others too many thereby reducing the efficiency of supplementation and wasting resources.

A second strategy is based on the renewal rate of the human intestinal mucosa (seven days) suggesting that it is possible to reduce anemia with weekly supplementation of iron. According to some studies this is as efficacious as daily supplementation in reducing the prevalence of anemia [7-9] although Engstrom et al. found that this is not be the case in 6- to 12-month-old infants [10]. Even so this regimen requires careful administration as missed doses should be replaced on the following day, a situation that is difficult to control.

As anemia is highly prevalent among children of developing countries including Brazil [11-13] and because of the negative effects of anemia on psychomotor development [14] different strategies should be investigated in order to reduce non-compliance to treatment.

The aim of this study was to assess the efficacy and feasibility of cyclical iron supplementation with the interval between supplementations being based on the mean life of red blood cells of approximately 120 days [15].

## Methods

This was a randomized controlled trial involving two intervention groups: Weekly and Cyclical Groups based on the type of supplementation.

The design of this study followed the ethical requirements established by Resolution 196/96 of the National Health Council of the Ministry of Health. The Research Ethics Committee of the Medicine School in São José do Rio Preto, Brazil (process # 0708/2006) approved the study, which was conducted from January 2007 to July 2008, in Bady Bassitt, a town in the State of São Paulo, Brazil.

The town of Bady Bassit was chosen as the setting for this study because of the possibility of enrolling the entire under five-year-old population attending all four government daycare centers in the town and because of the relatively similar background of all the children.

The subjects (252) were 24- to 59-month-old children from four local public daycare centers. All children attended the daycare centers from 7:00 a.m. to 5:00 p.m. and received the same type of food, which was provided by a central kitchen run by the local council. A typical daily diet at school included bread and butter or cheese, milk, biscuits and fruit for breakfast, rice, beans, meat (minced beef, chicken, liver or fish), vegetables and a fruit or jelly for lunch and bread and butter or cheese, biscuits and fruit juice before going home.

Two weeks prior to iron supplementation, all the children in the study had parasitological tests with stool (feces) analysis. Those who presented with infections at the beginning of the intervention, reported any infection within two weeks before the supplementation, presented with hereditary anemia or those who were already taking medication containing ferrous sulfate were excluded from the study.

Thus, of the 252 children who were attending the daycare centers, only 131 parents consented to permit their children participate in the study. Of these 11 were excluded, six due to infections, four were already taking medicines containing ferrous sulfate and one had hereditary anemia.

Hence, iron supplementation was started in 110 anemic and non-anemic children. The children were randomly assigned (table of randomly distributed numbers) to one of two intervention groups with 55 subjects each. Sample size calculations indicated that with ≥ 33 children per group, it would be possible to detect hemoglobin changes with a power of 0.8 and p-value = 0.05, considering a prevalence of anemia of around 40% and a 50% reduction.

During the study, 11 children left the daycare centers and the study. Thus, the data discussed in this paper is on 99 children that received all the iron doses. Iron supplementation was administered to the children on empty stomachs after they had been playing for a short time so that they better accepted the medication by staff of the daycare centers who also recorded compliance to treatment.

Codes were used to identify bottles of unflavored elemental Fe (ferrous sulfate) oral solution (30 mL) in both groups. The Fundação Remédio Popular, a laboratory kept by the government of São Paulo State, provided the medication, with one drop containing 1 mg elemental iron.

Children in the Weekly Group received a single 30 mg oral dose (30 drops – 1.5 mL) ferrous sulfate using a 2-mL disposable syringe every Wednesday during the ten months of the study. Children in the Cyclical Group received a daily dose of 30 mg of elemental Fe on 20 consecutive school days (one month) repeated after four months without medication.

If a child in the Weekly Group missed a dose due to absence from the daycare center, the missed dose was administered on the day of the child’s return to the daycare center. Children in the Cyclical Group who were absent during the supplementation period continued to receive iron upon their return to the daycare center until all 20 scheduled doses had been administered. Therefore, the duration of supplementation (10 months) was the same for both groups and all children received the same number of doses (40).

In order to compare the groups, anthropometric measurements were taken of all children prior to the intervention including weight (weighing scales: scale 50 g), height (unstretchable measuring tape: scale 0.1 cm) and age. Measurements were made by a single trained nurse. Moreover, venous blood samples were drawn for laboratory tests to quantify the serum hemoglobin of each child at the start and at the end of the experimental period. Hemoglobin concentrations were calculated using a Coulter STKS ^®^ device (Coulter Electronics, Inc. Hialeah, Florida U.S.A.) which uses the cyanomethemoglobin method of analysis [16].

If a child still presented with anemia at the end of the study, he/she was referred for specialist treatment. Anemia in this age range means hemoglobin concentration levels lower than 11.0 g/dL [1].

At the end of the experimental period, the four staff members of the four daycare centers who had administered the medication answered the following question: “In your opinion, which of the two treatments was simpler to administer and why?”

### Statistical analysis

The t-test for paired samples was used to compare means between groups, and the normal approximation test for two ratios or the Fisher’s exact test, as recommended, were used to compare event probability in both groups. Statistical analysis was performed using the Minitab computer program version 12.22 (Minitab Inc^®^) and the adopted level of significance was 5% (p-value < 0.05).

## Results

The parasitological examinations before the start of supplementation did not detect helminthes in any of the participants.

Data revealed that both groups were comparable in terms of age, height and weight before iron supplementation (p-value > 0.05 – Table 1) and also in terms of hemoglobin concentration (p-value = 0.56).

**Table 1:** Characteristics of the 99 children of the weekly and cyclical groups at the start of the study

**Characteristic**	**Weekly Group (n = 51)**	**Cyclical Group (n = 48)**	**p-value**
Mean age - months	44.44 ± 9.19	46.53 ± 10.32	0.30
Weight – Kg	16.10 ± 2.57	16.19 ± 2.67	0.87
Height – m	1.00 ± 0.07	1.01 ± 0.08	0.97
Gender - male (%)	49.21	58.33	0.41

Data shown as means ± standard deviation or percentage.

Immediately after receiving the medication, the staff member of the daycare center who administered the iron asked the children if they had experienced any side effects due to the medication (e.g. nausea, stomach pain, diarrhea, constipation). During the experimental period, three children (one in the Weekly Group and two in the Cyclical Group: 3.03% of the total) suffered from nausea probably due to the medication. However, they remained in the study until the end of the intervention period.

Overall, the percentage of anemic children (hemoglobin < 11.0 g/dL) decreased from 20.20% at the start of the study to 5.05% at the end (p-value = 0.0005). The prevalence of anemia in the Weekly Group (n = 51) decreased from 17.65% to 3.92% (p-value < 0.0005) and for the Cyclical Group (n = 48) the prevalence of anemia decreased from 22.90% to 6.25% (p-value < 0.0005). Thus the prevalence of anemia decreased significantly in both groups (p-value < 0.0005), however the results were not significantly different between groups (p-value = 0.35).

After 10 months of supplementation, there were statistically significant increases in blood hemoglobin concentrations for both groups (Table 2). An overall increase in the mean hemoglobin concentration of 0.37 g/dL was observed (p-value = 0.001).

**Table 2:** Initial and final mean blood hemoglobin concentrations for both groups of children on the different protocols of iron supplementation

**Group**	**Anemia (%)**		**Hemoglobin (g/dL)**			
	**Initial**	**Final**	**Initial**	**Final**	**Change**	**p-value**
The Weekly Group (n = 51)	17.65	3.92	11.75 ± 0.76	12.02 ± 0.72	0.27 ± 0.77	= 0.016
The Cyclical Group (n = 48)	22.90	6.25	11.66 ± 0.91	12.13 ± 0.89	0.47 ± 0.72	< 0.0005

Data shown as means ± standard deviation.

After 10 months of supplementation, the increases in hemoglobin concentration were not significantly different between the groups (p-value = 0.17). All the staff members of the daycare centers stated that the cyclical regimen was easier in respect to dose administration and treatment management in comparison with weekly supplementation.

## Discussion

A significant reduction in the prevalence of anemia and increase in the mean hemoglobin concentration were found after supplementation in cycles (Cyclical Group). This occurred with two short periods of daily iron administration; both the duration (20 days) of supplementation and the medication dose were smaller than reported in other studies [7,8,17].

Likewise, for the group that received weekly supplementation (Weekly Group), a significant reduction in the prevalence of anemia and increase in mean hemoglobin concentration resulting from a proportionally lower weekly dosage of iron compared to that of other studies employing the same procedure was observed [7,8,17].

Both approaches in the present study reduced the side effects associated to the ingestion of elemental iron compared to the administration of high dosages or longer periods of supplementation [7,8,17]. This reduction favored high compliance and no children left the study due to the treatment itself.

Regarding the feasibility of the two strategies, the staff members of the daycare centers reported greater difficulty in administering and managing the medication in the weekly group. When children missed daycare, their absence was recorded on individual charts, and the staff members were careful to administer the missing dose on the day of the child’s return.

According to the daycare staff, it was easier and less time-consuming to manage the group submitted to cyclical supplementation. On their return after absence from daycare, these children received normal medication and then continued to receive the missed doses at the end of the supplementation until 20 doses of iron supplementation had been administered. As this strategy only involved two cycles, extra medication was only needed twice throughout the ten months of intervention.

Iron supplementation is the choice treatment for populations with high prevalence of anemia [1]. According to previous studies, iron deficiency is the main cause of anemia in preschoolers [7,8,11,13,17,18]. Because our goal was to assess the reduction in the prevalence of anemia, the blood hemoglobin concentration was chosen as the laboratory test [11,13,18]. It is important to remember that iron deficiency is not the only cause of anemia and so iron supplementation will not resolve all cases.

The strategy proposed in this study involved short periods of daily supplementation repeated at intervals based on the mean life of red blood cells which is approximately 120 days [15]. We assessed the efficacy of this alternative procedure by comparing it with intermittent supplementation (weekly). Several previous publications report on the positive results of the weekly approach [7,8,13,17,18].

This study has indicated that the supplementation in cycles significantly reduces the prevalence of anemia in preschoolers. One limitation of this study, as in most clinical trials, is that the researchers are more able to control the behavior of participants and so compliance tends to be much better than in everyday situations. However this does not invalidate the results of the study as the comparisons made here are with other studies and the authors believe that this technique of cyclical supplementation will improve overall compliance.

## Conclusion

Both regimens significantly reduced the prevalence of anemia among preschoolers, but the findings reveal cyclical supplementation was easier to administer and control. Therefore, cyclical iron supplementation represents a new strategy to control anemia among preschoolers. Further studies can help assess the use of this strategy in larger populations and in a non-supervised setting would be of great interest.

## Competing interests

The authors declare that there is no competing of interests.

## Authors’ contributions

GGPLC contributed to the study design, the fieldwork, data collection, interpretation of results, drafting of the manuscript, writing and editing it. PMC contributed to the study design, statistical analysis, interpretation of data and drafting of the manuscript, writing and editing it. JAC contributed to the study design, statistical analysis, interpreted the results and drafting of the manuscript. All authors read and approved the final manuscript.

## Pre-publication history

The pre-publication history for this paper can be accessed here:

http://www.biomedcentral.com/1471-2458/13/21/prepub
